# TFAP2A potentiates lung adenocarcinoma metastasis by a novel miR-16 family/TFAP2A/PSG9/TGF-β signaling pathway

**DOI:** 10.1038/s41419-021-03606-x

**Published:** 2021-04-06

**Authors:** Yanlu Xiong, Yangbo Feng, Jinbo Zhao, Jie Lei, Tianyun Qiao, Yongsheng Zhou, Qiang Lu, Tao Jiang, Lintao Jia, Yong Han

**Affiliations:** 1 Department of Thoracic Surgery, Tangdu Hospital, Fourth Military Medical University, Xi’an, China; 2 State Key Laboratory of Cancer Biology, Department of Biochemistry and Molecular Biology, Fourth Military Medical University, Xi’an, China; 3 Department of Thoracic Surgery, Air Force Medical Center, PLA, Beijing, China

**Keywords:** Non-small-cell lung cancer, Metastasis

## Abstract

Transcription factor AP-2α (TFAP2A) was previously regarded as a critical regulator during embryonic development, and its mediation in carcinogenesis has received intensive attention recently. However, its role in lung adenocarcinoma (LUAD) has not been fully elucidated. Here, we tried to investigate TFAP2A expression profiling, clinical significance, biological function and molecular underpinnings in LUAD. We proved LUAD possessed universal TFAP2A high expression, indicating a pervasively poorer prognosis in multiple independent datasets. Then we found TFAP2A was not indispensable for LUAD proliferation, and exogenous overexpression even caused repression. However, we found TFAP2A could potently promote LUAD metastasis possibly by triggering epithelial–mesenchymal transition (EMT) in vitro and in vivo. Furthermore, we demonstrated TFAP2A could transactivate Pregnancy-specific glycoprotein 9 (PSG9) to enhance transforming growth factor β (TGF-β)-triggering EMT in LUAD. Meanwhile, we discovered suppressed post-transcriptional silencing of miR-16 family upon TFAP2A partly contributed to TFAP2A upregulation in LUAD. In clinical specimens, we also validated cancer-regulating effect of miR-16 family/TFAP2A/PSG9 axis, especially for lymph node metastasis of LUAD. In conclusion, we demonstrated that TFAP2A could pivotally facilitate LUAD progression, possibly through a novel pro-metastasis signaling pathway (miR-16 family/TFAP2A/PSG9/ TGF-β).

## Introduction

Lung cancer possesses the highest mortality among malignancies worldwide, and lung adenocarcinoma (LUAD) ranks the main subtype^1,2^. In the past decades, advancement in oncogenic mechanisms has intensively improved therapeutic strategies of LUAD^3^. Molecular targeted therapy based on driver mutations and immune checkpoint blockade have both acquired surprisingly successes in patients with specific genetic or immune background^3,4^. Nevertheless, it is still too early to celebrate for the ultimate triumphs, since substantial patients exhibit primary insensitivity and even patients benefited initially could suffer inevitable resistance^4^. In-depth understanding of malignant mechanisms is urgently needed for LUAD.

Transcription factor AP-2α (TFAP2A) was previously thought to be a critical mediator in embryonic development, especially for the formation of neural crests and epidermis^5,6^. Recently, TFAP2A has been unveiled for pivotal function in carcinogenesis^7^. However, due to cancer heterogeneity and transcriptional diversity, TFAP2A has showed paradoxical effects in different tumors^7^. For example, in liver cancer, breast cancer, glioma and colon cancer, TFAP2A behaves more like a tumor suppressor, while in neuroblastoma, pancreatic cancer and leukemia, it shows oncogenic potential^7^.

In lung cancer, TFAP2A still exhibits contradictions. The initial reports suggested that TFAP2A played a cancer-inhibitory role for proliferation suppression, apoptosis induction and chemosensitivity enhancement^8^, as well as suppressive function in nicotine-derived carcinogenesis^9,10^, and potentiating effect for tumor suppressor human liver DnaJ-like protein (HLJ1)^11^. However, successive studies have heralded cancer-promoting functions of TFAP2A in lung cancer like transactivating heme oxygenase-1 (HO-1) to facilitate tumor growth^12^, upregulating telomerase to resist apoptosis^13^, as well as increasing keratin 16 (KRT16) and Inositol-Trisphosphate 3-Kinase A (ITPKA) to promote the proliferation, migration and invasion of lung cancer^14,15^.

In lung cancer, therefore, the role of TFAP2A has not been clearly clarified and its expression profile, clinical association, biological function and regulatory mechanism still calls for further investigation. Considering the principal contribution of LUAD towards lung cancer, we tried to explore detailed function of TFAP2A in LUAD. We discovered upregulated expression profile and conforming prognostic risk of TFAP2A in multiple LUAD datasets. And we demonstrated that TFAP2A could promote LUAD metastasis via a novel signaling axis (miR-16 family/TFAP2A /PSG9/TGF-β).

## Materials and methods

### LUAD datasets

LUAD datasets were acquired from The Cancer Genome Atlas (TCGA) program and Gene Expression Omnibus (GEO) database. And different dataset was utilized for different usage considering data characteristics respectively (Supplementary Table 1).

### Tissue microarray (TMA), immunohistochemistry (IHC) and evaluation

262 LUAD specimens (131 paired cancerous tissues and normal tissues) to construct LUAD-TMA were collected from Tangdu Hospital, the Fourth Military Medical University (Xi’an, China) in accordance with ethical approve. In brief, microarrays were dewaxed, antigen repaired, treated with hydrogen peroxide and blocked, then incubated with specific primary antibodies (TFAP2A: ab52222, Abcam, Cambridge, UK; E-cadherin: GB14076, Servicebio, Wuhan, China; N-cadherin: GB11135, Servicebio) and horseradish peroxidase (HRP)-labeled secondary antibody, followed by colored, counterstained and microscopy. IHC scoring (H) was used to evaluate protein expression level: H = Σ(pi*i), in which ‘pi’ represents the percentage of positive cells and ‘i’ represents the intensity.

### Cell lines

Human LUAD cell lines (PC-9, H1650 and HCC827) and HEK293T were purchased from the Cell Bank of the Chinese Academy of Sciences (Shanghai, China). Cells were cultured in RPMI-1640 or DMEM medium supplemented with 10% fetal bovine serum (FBS) at 37 °C in a humidified 5% CO_2_ atmosphere. All cells were authenticated by short tandem repeat profiling.

### Cell infection and transfection

For cell infection, recombinant lentiviruses (Hanbio, Shanghai, China) for gene interference and overexpression were induced into cells, followed by puromycin selection for stable cell models. For transcient transfection, siRNAs (RiboBio, Guangzhou, China), plasmids (Hanbio) and miRNA mimics (RiboBio) were induced into cells using Lipofectamine 2000 (Invitrogen, CA, USA) according to the manufacturer’s instructions. The utilized nucleic sequences used are listed in Supplementary Table 2.

### Quantitative reverse transcription-polymerase chain reaction (qRT-PCR)

Total cell RNA was extracted using RNAiso reagent (TaKaRa, Dalian, China) and cDNA was synthesized from 1.0 μg of total RNA using the PrimeScript RT Master Mix (TaKaRa). For miRNA, reverse transcription was performed using miDETECT A Track^TM^ miRNA qRT-PCR Starter Kit (RiboBio). Real-time PCR was performed using SYBR Premix Ex Taq II (TaKaRa) with a CFX96 Real-Time PCR detection system (Bio-Rad, CA, USA). β-actin and U6 were used as internal reference for mRNAs and miRNAs respectively. The 2^−ΔΔCt^ method was used to determine relative gene expression. The sequences of primers used are listed in Supplementary Table 2.

### Western blot

Proteins from cell lysates were separated by SDS-PAGE and transferred to a nitrocellulose membrane, which was incubated with primary specific antibodies (TFAP2A: ab108311/ab52222, Abcam; β-actin: #4970, CST, MA, USA; E-cadherin: #3195, CST; N-cadherin:#13116, CST; PSG9: GTX120479, GeneTex, CA, USA; Smad2: A18674, ABclonal, Wuhan, China; Phospho-Smad2^S465/467^: AP0548, ABclonal), followed by HRP-conjugated secondary antibody. ECL reagent (Merck Millipore, Darmstadt, Germany) was applied for protein detection.

### CCK8 proliferation assays

Cells in the logarithmic growth phase were fully mixed, inoculated in a 96-well plate, continuously cultured for 96 hours, and the number of living cells was measured every 24 hours with CCK8 kit (Dojindo, Kumamoto, Japan).

### Plate cloning experiments

Cells in the logarithmic growth stage were fully mixed into a single cell suspension, inoculated in a 6-well plate, stained with crystal violet after 2–3 weeks, and the number of cell clones was counted.

### Cell-cycle assays

Cells were washed with phosphate-buffered saline, fixed with cold 75% ethanol, incubated with RNase A, stained with propidium iodide, and then analyzed by flow cytometry.

### Transwell experiments

Place the Transwell chamber (Corning, NY, USA) into a 24-well plate and add the configured Matrigel (Corning). Cells were suspended in medium containing no FBS and seeded into the upper chamber, and culture medium containing 20% FBS was added to the lower chamber. After incubation for 12–24 h, the cells were fixed in methanol and stained with crystal violet. Cells resident on the lower surface of the filter were counted for statistical analyzing. Analogously, Transwell chambers without configured Matrigel (Corning) were used for cell migration examination.

### Wound-healing assays

Cell layers were carefully scratched by a 200-μL pipette tip to generate wounds, which were photographed after 0, 24 or 36 h to measure the widths for statistical analyzing.

### Metastasis assays in vivo

Cells transferred with recombinant lentiviruses containing firefly luciferase (Hanbio) were injected into the tail veins of 6-weeks-old NOD-*Prkdc*^*scid*^ *IL2rg*^*tm1*^/Bcgen (B-NDG) mice (Biocytogen, Beijing, China). After 3–4 weeks, mice were injected with D-Luciferin potassium salt by intraperitoneal injection, and photographed by fluorescence stereomicroscope under isoflurane anesthesia. After that, mice were sacrificed by isoflurane anesthesia, and metastatic nodes in lung specimens were counted after hematoxylin-eosin (H&E)–staining. Mice were maintained at specific pathogen-free conditions in Laboratory Animal Center of the Fourth Military Medical University. All animal experiments carried out were in accordance with ethical animal care.

### Luciferase reporter assays

Luciferase activity was determined in cell lysates using a dual-luciferase assay system (Promega, WI, USA). The Pregnancy-specific glycoprotein 9 (PSG9) promoter fragment (2000 upstream from the transcription start point of human PSG9 gene) was cloned into pGL3 vector (Promega) to establish pGL3-PSG9-Promoter-Luc plasmid. Fragments containing 3′-untranslated region (3′-UTR) of TFAP2A include complete putative targeting sites and five mutant targeting sites of miR-16 family, which were cloned into psiCHECK-2 vector (Promega) to constructed psiCHECK-2-TFAP2A-3′UTR-Luc-WT and psiCHECK-2-TFAP2A-3′UTR-Luc-Mut1/2/3/4/5 plasmids respectively. Meanwhile, pRL-TK plasmid (Promega) carrying renilla luciferase was co-transfected for normalization. The specific sequences are listed in the following Results parts.

### Chromatin immunoprecipitation (ChIP)

The ChIP assays were conducted according manufacturer’s protocol (The SimpleChIP® Plus Enzymatic Chromatin IP Kit: #9005, CST). Briefly, cells were fixed with formaldehyde, and the chromatin was fragmented by enzymatic digestion and sonication. Then precleared chromatin was immunoprecipitated overnight with specific antibodies (TFAP2A: 13019–3-AP, Proteintech, Wuhan, China; Normal Rabbit IgG: #2729, CST; Histone H3 (D2B12) XP® Rabbit mAb: #4620, CST). The enrichment of specific DNA fragments was analyzed by qPCR. The primers used are listed and explained in Supplementary Table 3.

### Statistical analysis

*P* < 0.05 was considered significant. R language offered packages for arithmetic functions^16^. Wilcoxon rank sum test, two-group *t*-test, ANOVA, and Dunnett *t*-test were utilized for differential analysis according to data characteristics. DESeq2 package was used for differential gene expression analysis. ROC analysis was applied to define the cut-off value for high or low expression. Cox regression model, Kaplan–Meier curve and log-rank test were used for survival analysis. Z-score was used to normalize data and Pearson correlation analysis was utilized for correlation assessment. The chi-square test was used for rates comparison.

## Results

### TFAP2A exhibits elevated expression profile in LUAD

**Fig. 1:**
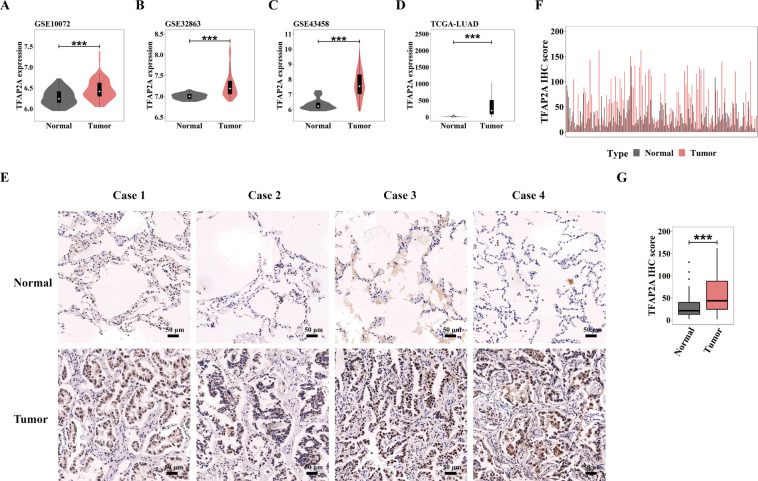
TFAP2A shows high expression profile in LUAD. **A**–**D** TFAP2A transcript levels between normal tissues and tumor tissues in LUAD datasets (GSE10072, GSE32863, GSE43458 and TCGA-LUAD). **E** IHC staining for TFAP2A protein levels in four paired normal tissues and tumor tissues in LUAD-TMA. **F** TFAP2A IHC scoring of 127 paired normal and tumor tissues in LUAD-TMA. **G** Statistical analysis of TFAP2A protein level (IHC scoring) in LUAD-TMA. *p < 0.05; **p < 0.01; ***p < 0.001. Data are presented through boxplots.

Preferentially, TFAP2A expression profiling needs clarifying in LUAD. We first explored the mRNA levels of TFAP2A in four transcriptional LUAD datasets (GSE10072, GSE32863, GSE43458 and TCGA-LUAD). We found that, compared with normal tissues, the TFAP2A mRNA levels in tumor tissues were all significantly increased (Fig. 1A–D). Then we built LUAD TMA containing 131 pairs of tumor/normal tissues. By immunohistochemistry scoring (127 contact pairs eventually retaining), we found protein expression of TFAP2A in tumor tissues was also up-regulated, mainly distributed in the nucleus (Fig. 1E), and statistically significant (Fig. 1F, G). These data clearly pointed out LUAD possessed high expression of TFAP2A, indicating its cancer-promoting potential.

### High TFAP2A expression could predict poor clinical prognosis in LUAD

**Fig. 2:**
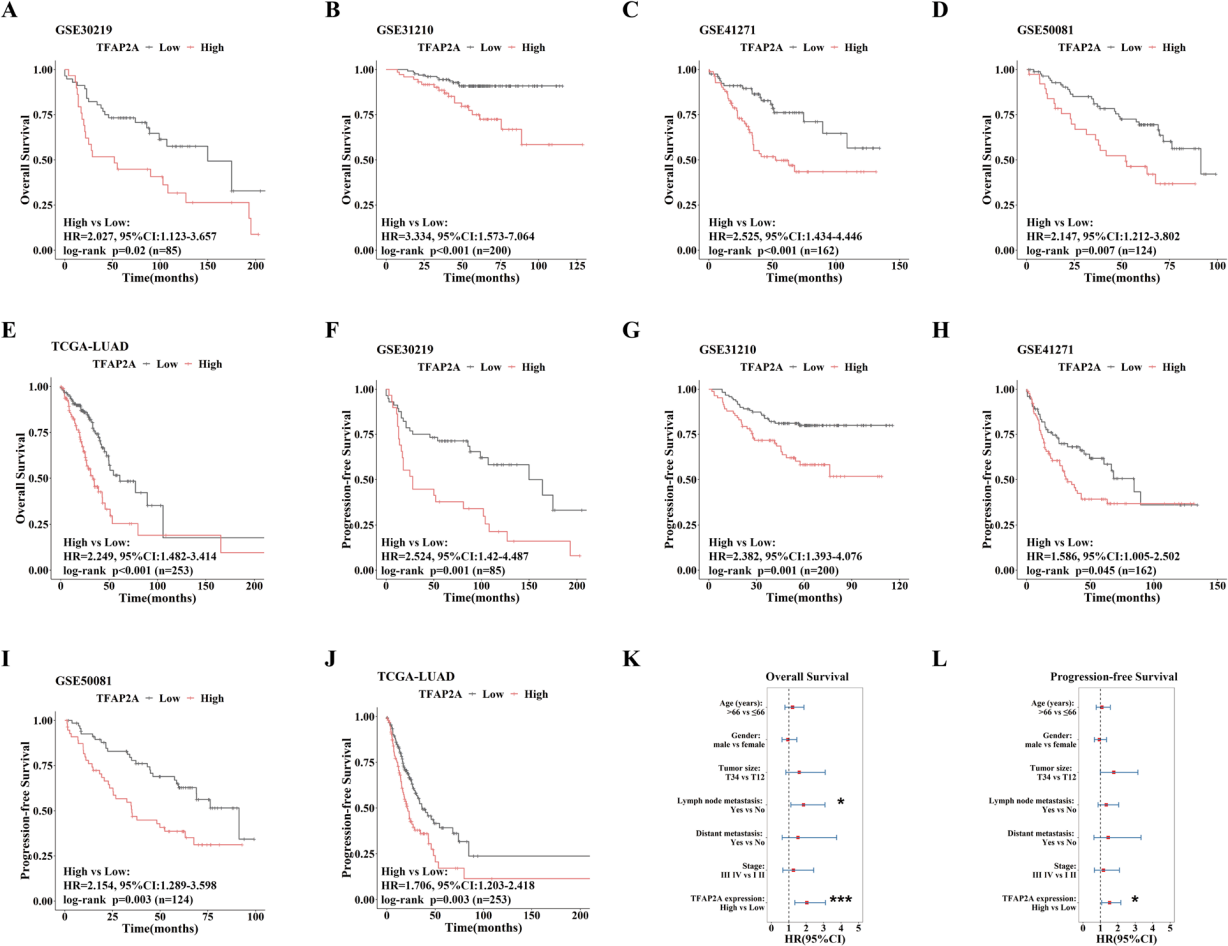
High expression of TFAP2A in LUAD predicts poor clinical prognosis. **A**–**E** OS curves between LUAD patients with high or low TFAP2A expression and HRs of univariate analysis (high group vs. low group) in five independent LUAD datasets (GSE30129, GSE31210, GSE41271, GSE50081 and TCGA-LUAD). **F**–**J** PFS curves between LUAD patients with high or low TFAP2A expression and HRs of univariate analysis (high group vs. low group) in five independent LUAD datasets (GSE30129, GSE31210, GSE41271, GSE50081 and TCGA-LUAD). **K**, **L** HRs of multivariate analysis (age, gender, TNM, clinical stage and TFAP2A expression) towards OS and PFS in TCGA-LUAD datasets. *p < 0.05; **p < 0.01; ***p < 0.001.

Then we investigated clinical significance of TFAP2A in LUAD. Five independent LUAD datasets (GSE30219, GSE31210, GSE41271, GSE50081 and TCGA-LUAD) were applied for survival analysis (ROC curve based on survival status was used to define high or low TFAP2A expression). We found that patients with high TFPA2A expression in the five datasets all had poorer Overall Survival (OS) (Fig. 2A–E), as well as shorter Progression-free Survival (PFS) (Fig. 2F–J). To avoid confounding, we performed multivariate Cox analysis to evaluate risk prediction of TFAP2A in TCGA-LUAD dataset for its relatively more complete clinical records. We found that high TFAP2A expression was an independent risk factor for both OS and PFS (Fig. 2K, L). The above results fully proved that high TFAP2A expression predicted poorer clinical prognosis of LUAD.

### TFAP2A is not requisite for LUAD proliferation, but prominently promotes invasion and migration of LUAD

**Fig. 3:**
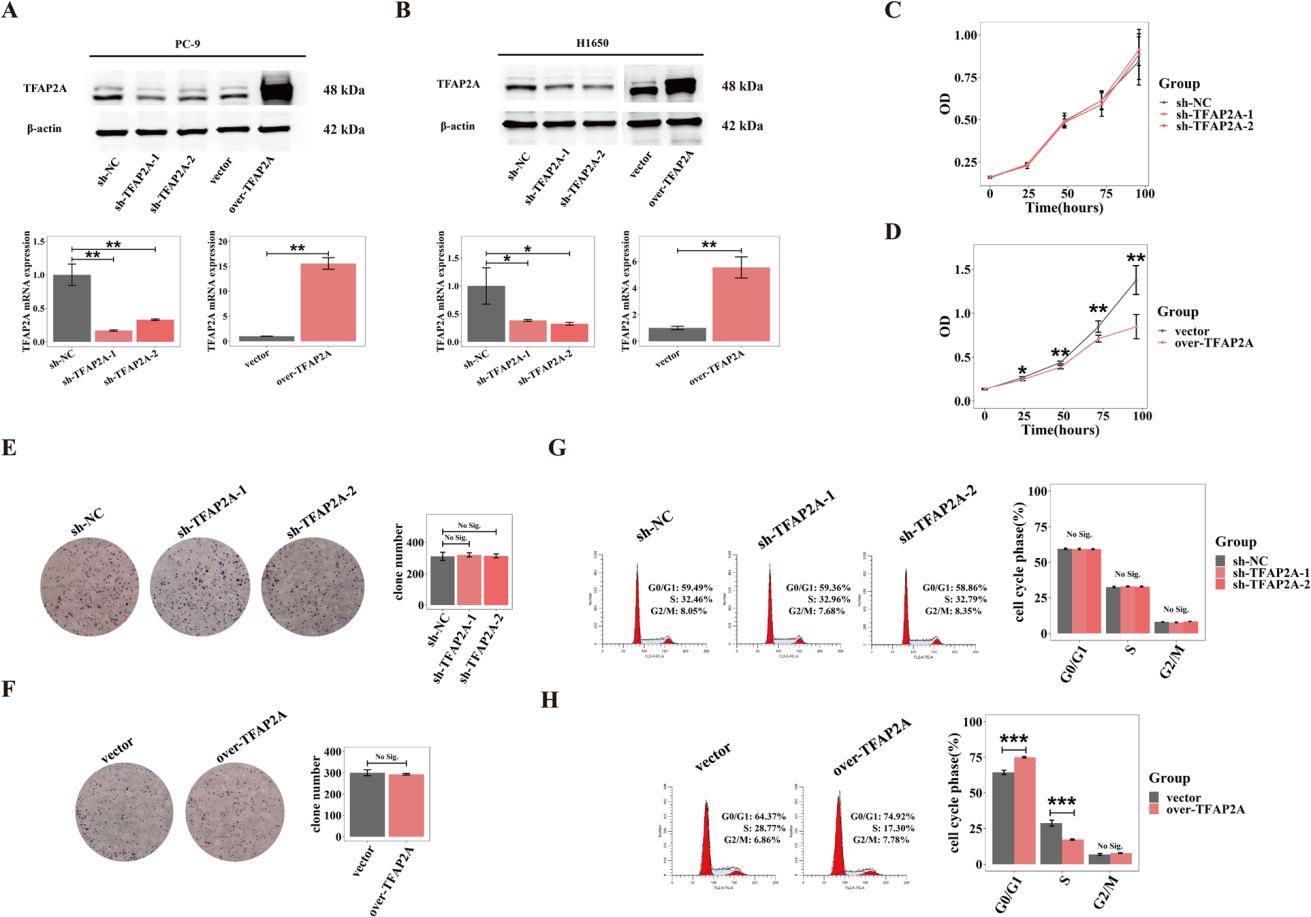
TFAP2A could not facilitate or even inhibit LUAD proliferation. **A**, **B** Protein expression (up) and transcript levels (down) of TFAP2A for TFAP2A knockdown and overexpression cell models of PC-9 (**A**) and H1650 (**B**) (stable gene interference and overexpression by recombinant lentiviruses). **C**, **D** Proliferation curves assessed by CCK8 assay during 96 h for TFAP2A knockdown and overexpression cell models of PC-9 (**C**, knockdown vs. control; **D**, overexpression vs. control). **E**, **F** Plate cloning and statistical analysis for TFAP2A knockdown and overexpression cell models of PC-9 (**E**, knockdown vs. control; **F**, overexpression vs. control). **G**, **H** Effects on cell cycle progression for TFAP2A knockdown and overexpression cell models of PC-9 (**G**, knockdown vs. control; **H**, overexpression vs. control). *p < 0.05; **p < 0.01; ***p < 0.001. *N*≥3, Data are presented as mean ± standard deviation (SD).

Next, we explored the biological effects of TFAP2A upon LUAD. We successfully established stable cell models of TFAP2A knock-down and overexpression in two LUAD cell lines (PC-9 and H1650) by recombinant lentiviruses infection (Fig. 3A, B). Uncontrolled proliferation remains the preliminary characteristic for cancer, we first investigated effect of TFAP2A upon LUAD proliferation. We found that TFAP2A knockdown did not affect proliferative activity of PC-9 cells (Fig. 3C), while exogenous TFAP2A overexpression inhibited proliferative activity of PC-9 cells (Fig. 3D), and TFAP2A had no significant effect on the cloning ability of PC-9 cells whether TFAP2A was knocked down or exogenously overexpressed (Fig. 3E, F). Further, we also found TFAP2A knockdown had little impact upon cell cycle progression of PC-9 cells (Fig. 3G), but TFAP2A exogenous overexpression could hold back cell cycle progression of PC-9 cells (Fig. 3H). These results possibly indicated that TFAP2A was not pre-requisite for LUAD proliferation, and exogenous TFAP2A overexpression could even restrain proliferation in cultured LUAD cells.

**Fig. 4:**
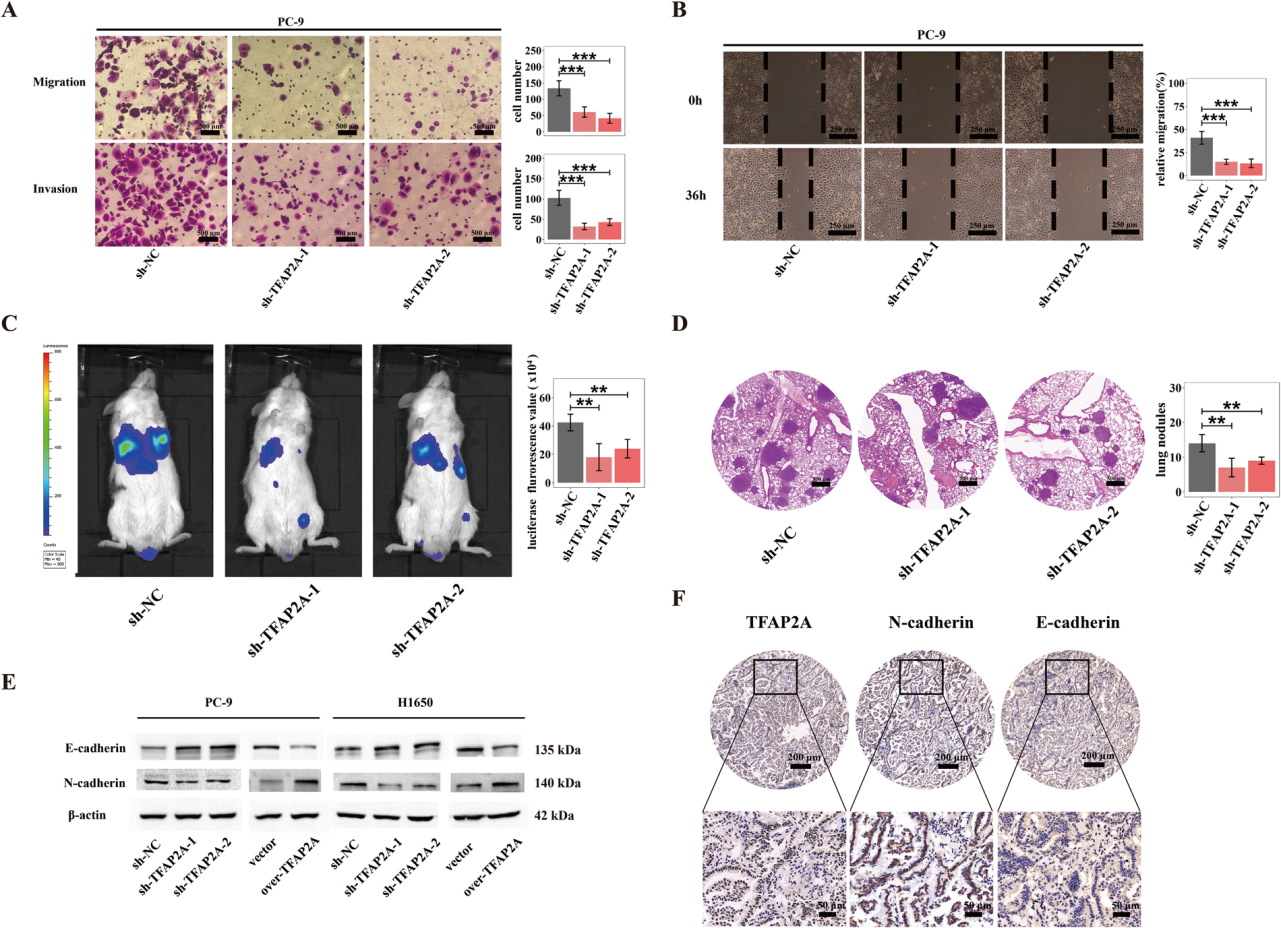
TFAP2A could markedly potentiate LUAD metastasis possibly by promoting EMT. **A** Cell migratory and invasive ability (left) by transwell assay as well as statistical analysis (right) for TFAP2A knockdown cell models of PC-9. **B** Cell migratory ability via wound-healing assay (left) and statistical analysis (right) for TFAP2A knockdown cell models of PC-9. **C** Fluorescence intensity detected in mice (left) and statistical analysis (right) for evaluation of vivo metastasis (five mice in each group). **D** H&E staining (left) and statistical analysis (right) for metastatic nodules in dissected lung specimen from mice (five mice in each group). **E** E-cadherin and N-cadherin protein levels for TFAP2A knockdown and overexpression cell models of PC-9 and H1650 respectively. **F** IHC staining for protein levels of TFAP2A, N-cadherin and E-cadherin in clinical specimens of LUAD (up, low resolution; down, high resolution). *p < 0.05; **p < 0.01; ***p < 0.001. *N*≥3, Data are presented as mean ± SD.

Metastasis is regarded as a core hallmark for cancer, also a prototypical indictor for malignant progression. Using transwell assays and wound-healing assays, we found that TFAP2A knockdown reduced cell invasion and migration in PC-9 cells (Fig. 4A, B), while TFAP2A overexpression significantly promoted metastatic capability of PC-9 cells (Fig. S1A, B). Meanwhile, similar conclusions were deduced in H1650 cells (Fig. S1C–F). Subsequently, through mouse tail vein injection of TFAP2A knockdown or control H1650 cells transferred with luciferase lentiviruses, we found TFAP2A knockdown significantly reduced vivo metastasis of LUAD. (Fig. 4C, D). Epithelial-mesenchymal transition (EMT) accounts largely for enhancing migratory and invasive ability of neoplastic cells, while upregulated N-cadherin and downregulated E-cadherin are canonical biomarkers indicating cell undergoing EMT. We found that TFAP2A could promote N-cadherin expression, and decrease E-cadherin expression in PC-9 and H1650 cells (Fig. 4E). Moreover, we found that clinical specimens of LUAD also exhibited high protein expression of both TFAP2A and N-cadherin, as well as low expression of E-cadherin (Fig. 4F). In summary, the above results demonstrated that TFAP2A could enhance metastatic potential of LUAD possibly by facilitating EMT.

### TFAP2A transactivates PSG9 to potentiate TGF-β signals-triggered metastasis

**Fig. 5:**
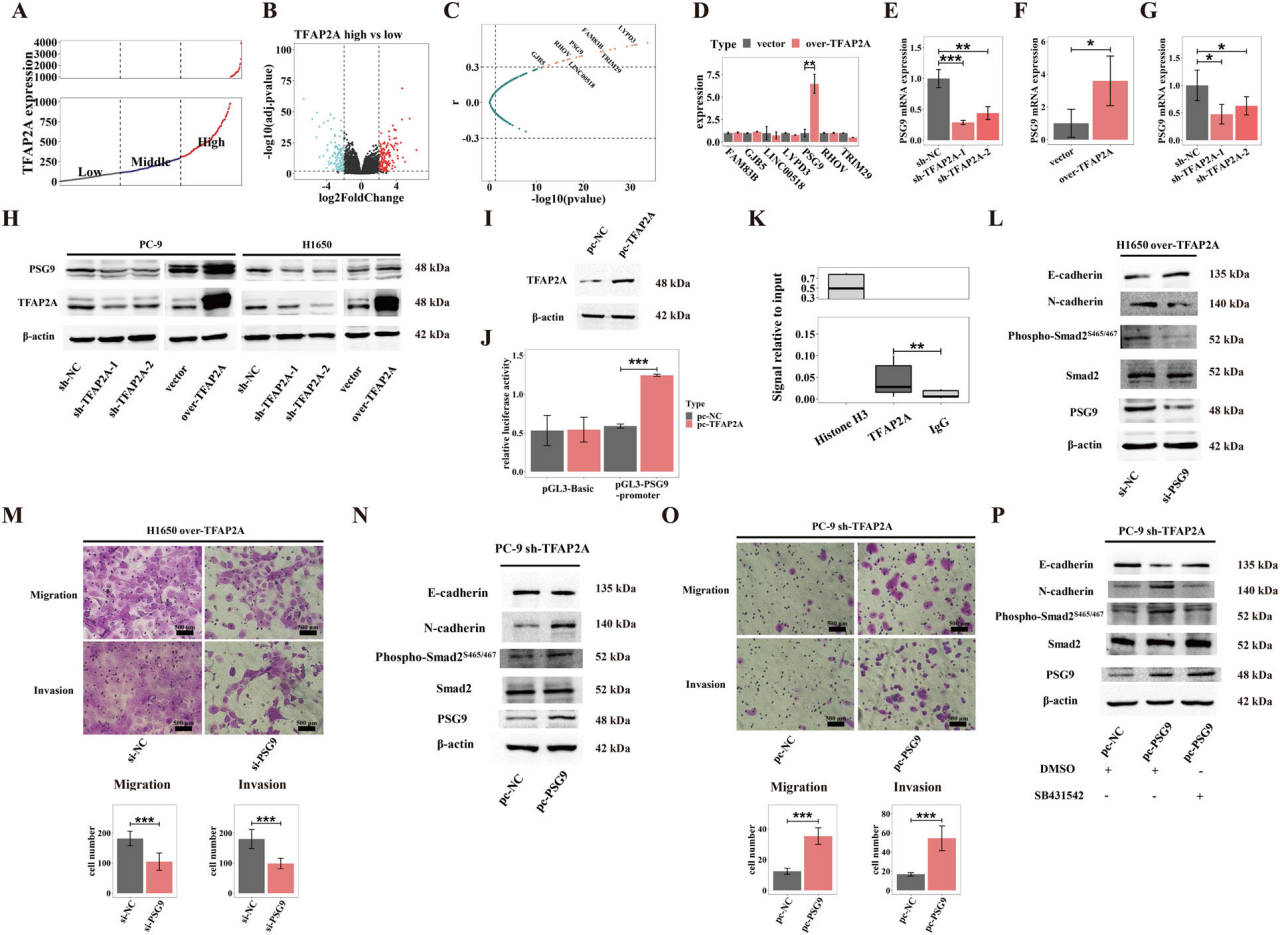
TFAP2A promotes LUAD metastasis by transactivating PSG9 to potentiate TGF-β signaling. **A** 515 Patients in TCGA-LUAD dataset were divided equably into high, middle and low group based on TFAP2A expression. **B** Volcano plot exhibiting differentially expressed genes (DEGs) between TFAP2A high and low expression groups. **C** *p*-values and coefficients(r) of expression correlation analysis between TFAP2A and DEGs described in **B**, DEGs with *p* < 0.05 and *r*>0.3 or *r* < -0.3 were regarded significant, further DEGs with TFAP2A-ChIP-Seq evidence in hTFtarget database were picked out and texted. **D** Transcripts levels of seven identified possibly TFAP2A-targeted genes in TFAP2A overexpression cell models of PC-9. **E** PSG9 transcript levels in TFAP2A knockdown cell models of PC-9. **F**, **G** PSG9 transcript levels in TFAP2A overexpression and knockdown cell models of H1650 (**F**, overexpression vs. control; **G**, knockdown vs. control). **H** TFAP2A and PSG9 protein levels for TFAP2A knockdown and overexpression cell models of PC-9 (left) and H1650 (right). **I** TFAP2A protein expression for TFAP2A overexpression cell models of HEK293T. **J** Effects for TFAP2A overexpression upon luciferase activity in HEK293T transfected with pGL3-Basic and pGL3-PSG9-promoter luciferase reporter (pc-TFAP2A vs. pc-NC). **K** Relative levels of immunoprecipitated PSG9 promoter regions (compared to Input chromatin) among positive control (Anti-Histone 3), negative control (Anti-IgG) and experiment group (Anti-TFAP2A) detected by qPCR. **L** EMT markers (E-cadherin/N-cadherin protein expression) and TGF-β signaling status (Phospho-Smad2^S465/467^/Smad2 protein expression) between PSG9 knockdown (si-PSG9) and control (si-NC) H1650 with stable TFAP2A overexpression. **M** Migratory and invasive abilities between PSG9 knockdown (si-PSG9) and control (si-NC) H1650 with stable TFAP2A over-expression. **N** EMT markers (E-cadherin/ N-cadherin protein expression) and TGF-β signaling status (Phospho-Smad2^S465/467^/Smad2 protein expression) between PSG9 overexpression (pc-PSG9) and control (pc-NC) PC-9 with stable TFAP2A knockdown. **O** Migratory and invasive abilities between PSG9 overexpression (pc-PSG9) and control (pc-NC) PC-9 with stable TFAP2A knockdown. **P** EMT markers (E-cadherin/ N-cadherin protein expression) and TGF-β signaling status (Phospho-Smad2^S465/467^/Smad2 protein expression) among control (pc-NC + DMSO), PSG9 overexpression (pc-PSG9 + DMSO), and PSG9 overexpression treated with TGF-β inhibitor (pc-PSG9 + SB431542) PC-9 with stable TFAP2A knockdown. *p < 0.05; **p < 0.01; ***p < 0.001. N≥3, Data are presented as mean ± SD or through boxplots.

TFAP2A mainly functions as a transcription factor, so its cancer-mediating effect largely attributes to critical transcriptional targets. First, we tried to primarily screen out targets of TFAP2A in LUAD according to gene expression correlations and public transcriptional database. According to TFAP2A expression of tumor tissues, we divided patients of TCGA-LUAD dataset into three groups: high, middle and low group (Fig. 5A). Then we initially identified a cohort of genes via differential gene expression analysis (high vs. low TFAP2A group) (Fig. 5B). Subsequently, by filtrating through correlation matrix of TFAP2A with its potential targets (*p* < 0.05, *r*>0.3 or *r* < −0.3), and further validating in hTFtarget database (ChIP-Seq evidence), we finally obtained 7 genes that TFAP2A possibly transactivates in LUAD (Fig. 5C).

Next, we validated 7 targets by stable cell models of TFAP2A knockdown or overexpression. We primarily used PC-9 models of TFAP2A overexpression, and found that only PSG9 was consistent with our prediction, that is TFAP2A overexpression significantly elevated mRNA level of PSG9 (Fig. 5D). We further examined PC-9 models with TFAP2A knockdown, and found that TFAP2A downregulation significant declined mRNA level of PSG9 (Fig. 5E). Then we corroborated that TFAP2A significantly increased the PSG9 transcription in H1650 models of TFAP2A overexpression and knockdown (Fig. 5F, G). Later, we confirmed that TFAP2A enhanced the protein level of PSG9 in both PC-9 and H1650 models of TFAP2A overexpression and knockdown (Fig. 5H). We then tried to prove the directly transcriptional activation of TFAP2A upon PSG9. We overexpressed TFAP2A in HEK293T cells (Fig. 5I), and found that TFAP2A overexpression was able to significantly increase firefly luciferase expression, when HEK293T was co-transfected with TFAP2A overexpression plasmids and constructed pGL3-PSG9-Promoter-Luc plasmids (Fig. 5J). Furthermore, ChIP assay also demonstrated TFAP2A could bind to promoter region of PSG9 (Fig. 5K). These results suggested that TFAP2A could directly transactivate PSG9.

PSG9 has been reported to effectively activate transforming growth factor β (TGF-β)^17^, which could pivotally trigger EMT^18^, indicating possible potential of PSG9 to facilitate malignant metastasis. We used rescue experiments to directly test whether PSG9 could undertake metastasis promotion of TFAP2A. On the one hand, we silenced PSG9 in TFAP2A overexpression H1650 models, we found that PSG9 inhibition could attenuate EMT and TGF-β activation, as well as decline the enhanced migration and invasion of LUAD caused by TFAP2A overexpression (Fig. 5L, M). On the other hand, we overexpressed PSG9 in TFAP2A knockdown PC-9 models, and found that PSG9 overexpression could partly recover EMT and TGF-β activation, along with compensation for the declined migration and invasion of LUAD induced by TFAP2A inhibition (Fig. 5N, O). Furthermore, we found that PSG9 promoted TFAP2A-enhanced EMT in LUAD cells, while the inhibitor of TGF-β signaling (SB431542, TargetMol, MA, USA) attenuated the EMT-promoting effect of PSG9 (Fig. 5P). Therefore, the above evidence suggested that TFAP2A promoted invasion and migration of LUAD possibly by transactivating PSG9 to potentiate TGF-β signaling pathways.

### Attenuated post-transcriptional silencing of miR-16 family upon TFAP2A contributes to upregulated TFAP2A levels in LUAD

**Fig. 6:**
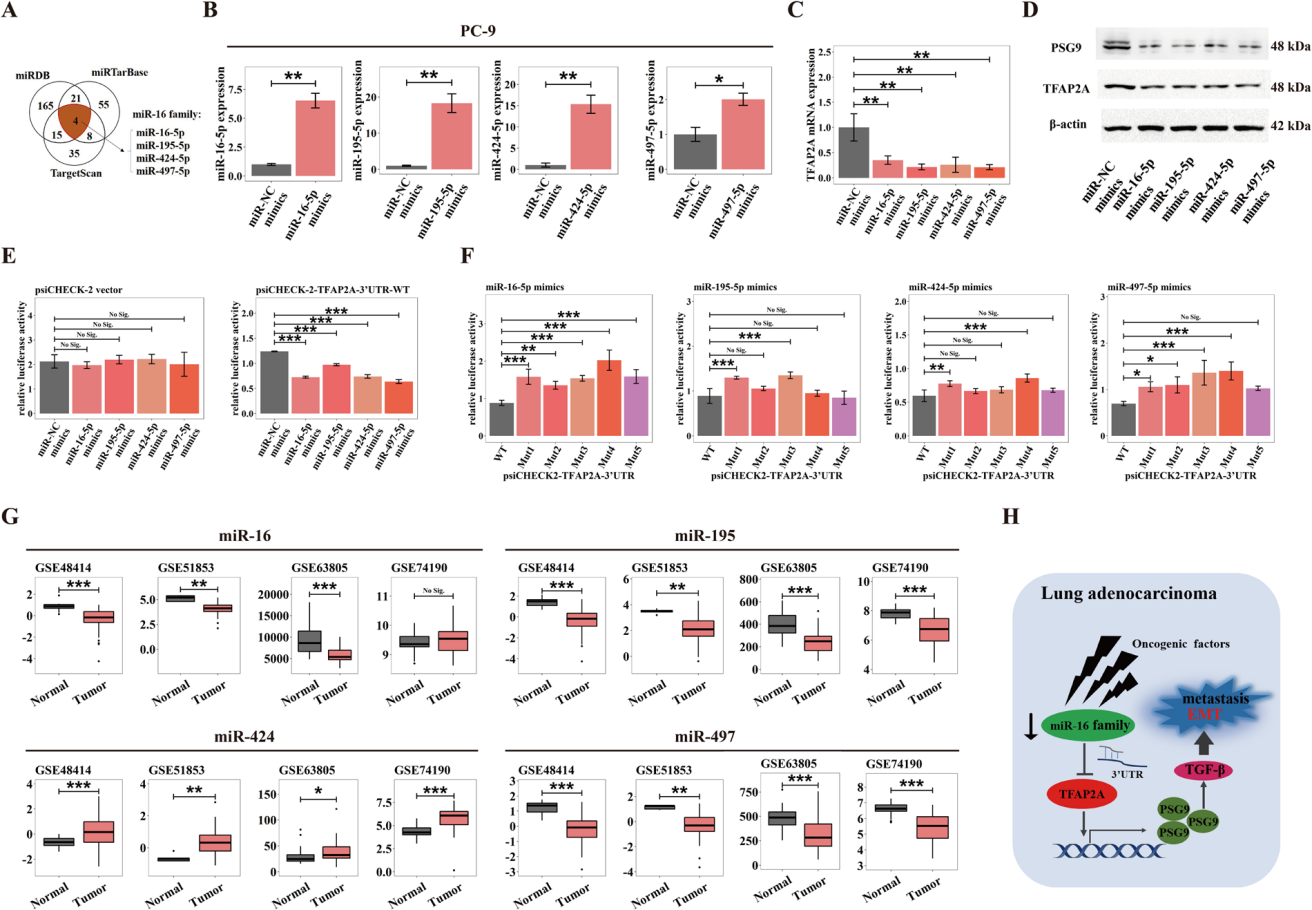
Post-transcriptional silencing of TFAP2A by miR-16 family is attenuated in LUAD. **A** Venn diagram exhibiting miRNAs possibly regulating TFAP2A via bioinformatic prediction from three miRNA database (miRDB, miRTarBase and TargetScan). **B** Overexpression efficacy for miR-16/195/424/497-5p mimics in PC-9. **C** Influence on TFAP2A transcript levels of miR-16/195/424/497-5p mimics transfection in PC-9. **D** Influence on TFAP2A and PSG9 protein levels of miR-16/195/424/497-5p mimics transfection in PC-9. **E** Dual luciferase assays of HEK293T co-transfected with miR-16/195/424/497-5p mimics and psiCHECK-2-TFAP2A-3′UTR-WT luciferase reporter (right) or control psiCHECK-2 vector (left). **F** Dual luciferase assays of HEK293T co-transfected with miR-16/195/424/497-5p, psiCHECK-2-TFAP2A-3′UTR-WT (WT) and psiCHECK-2-TFAP2A-3′UTR-Mut1/2/3/4/5 (Mut) luciferase reporters. **G** miR-16/195/424/497 levels between tumors and normal tissues in four LUAD datasets (GSE48414, GSE51853, GSE63805 and GSE74190). **H** In LUAD, multiple oncogenic factors induce miR-16 family suppression, thus hindering TFAP2A from post-transcriptional silencing by miR-16 family, therefore increased TFAP2A transactivates PSG9 to facilitate TGF-β signaling-triggered EMT and subsequent metastasis. *p < 0.05; **p < 0.01; ***p < 0.001. N≥3, Data are presented as mean ± SD or through boxplots.

The reason accounting for aberrant TFAP2A expression in LUAD is critical either. Posttranscriptional modification caused by miRNAs has a considerable impact upon gene expression regulation. By intersection analysis of three miRNAs database (miRDB, miRTarBase and TargetScan), we inferred that miR-16 family (miR-16-5p/miR-195-5p/miR-424-5p/miR-497-5p) could target at 3′UTR of TFAP2A through seed region (5′-AGCAGCA-3′) (Fig. 6A). By transfecting corresponding miRNA mimics into PC-9 cells, we found that the miR-16 family significantly down-regulated the mRNA and protein levels of TFAP2A and PSG9 (Fig. 6B–D). Similarly, LUAD cell lines H1650 and HCC827 also exhibited the same conclusion (Fig. S2A–F). According to TargetScan prediction, we constructed the wild-type (psiCHECK2-TFAP2A-3′UTR-WT) and five mutant types (psiCHECK2-TFAP2A-3′UTR-Mut) of luciferase reporter plasmids (Fig. S2G). Further, in HEK293T cells, we found that the miR-16 family could all decrease the luciferase activity of the psiCHECK2-TFAP2A-3′UTR-WT (Fig. 6E), but the specific binding sites of each member in miR-16 family were not in full accord (Fig. 6F). It has been reported diverse tumors possess downregulated miR-16 family, indicting possibly tumor suppressive capability. We investigated miR-16 family expression in LUAD by analyzing four independent datasets (GSE48414, GSE51853, GSE63805 and GSE74190), and found that miR-16/195/497 presented low expression in tumor tissues, while miR-424 showed high expression in tumor tissues, compared to normal tissues (Fig. 6G). This suggests that contributing proportions of specific members in miR-16 family upon TFAP2A need further clarifying, as well as other factors affecting TFAP2A content in LUAD. In brief, we deduced in LUAD, miR-16 family suppression caused by multiple oncogenic factors could liberate post-transcriptional silencing of TFAP2A, thus elevated TFAP2A could transcriptionally activate PSG9 to potentiate TGF-β signaling pathways, triggering EMT and subsequent metastasis (Fig. 6H).

### Clinical significance of miR-16 family/TFAP2A/PSG9 axis exists in LUAD

**Fig. 7:**
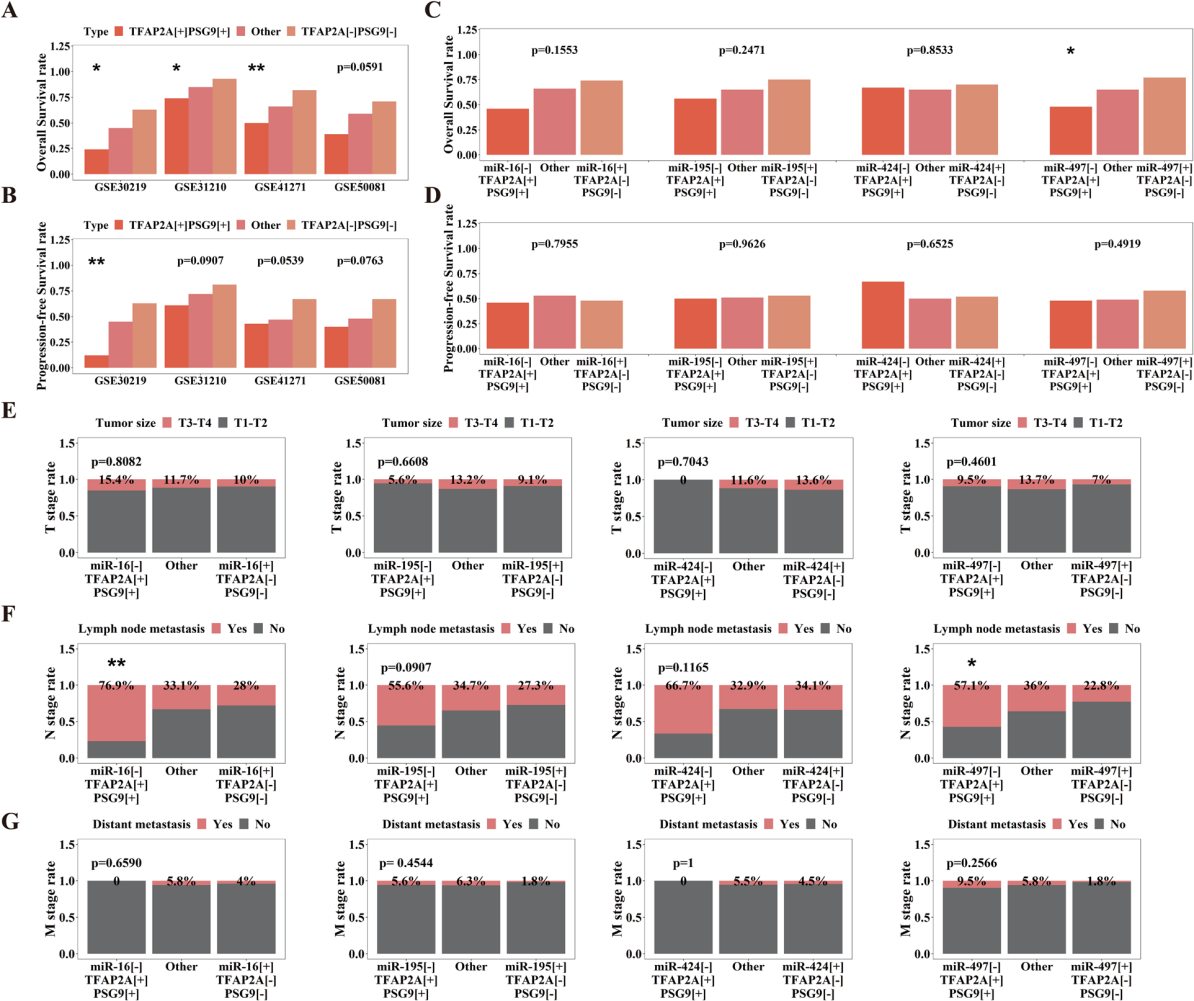
The miR-16 family/TFAP2A/PSG9 axis exhibits clinical effects upon LUAD. **A**, **B** The effects of TFAP2A/PSG9 on survival rate in four independent LUAD datasets (GSE30219, GSE31210, GSE41271 and GSE50081) (**A**, OS rate; **B**, PFS rate). **C**, **D** The influence of miR-16 family/TFAP2A/PSG9 upon survival rate in TCGA-LUAD dataset (**C**, OS rate; **D**, PFS rate). **E**–**G** The influence of miR-16 family/TFAP2A/PSG9 upon TNM parameters in TCGA-LUAD dataset (**E**, T parameters: tumor size; **F**, N parameters: lymph node metastasis; **G**, M parameters: distant metastasis). *p < 0.05; **p < 0.01; ***p < 0.001.

Finally, we investigated the clinical significance of the miR-16 family/TFAP2A/ PSG9 axis. First, we assessed the impact of the TFAP2A/PSG9 combination on survival rate by comparing patients of three groups divided by gene expression level (TFAP2A^+^ PSG9^+^, TFAP2A^-^ PSG9^-^, and Other) in four LUAD clinical datasets (GSE30219, GSE31210, GSE41271 and GSE50081). We found that TFAP2A/PSG9 was significantly related to OS rate, that is TFAP2A^+^ PSG9^+^ group had the lowest survival rate, while TFAP2A^-^ PSG9^-^ group possessed the highest OS rate, and statistical significance was exhibited in three datasets, and *p* value of GSE50081 was 0.0591 (Fig. 7A). We also found that TFAP2A^+^ PSG9^+^ group had the lowest PFS rate, and TFAP2A^-^ PSG9^-^ group possessed the highest PFS rate. It is worth noting that the difference of GSE30219 had statistical significance, while p values of GSE31210, GSE41271 and GSE50081 were 0.0907, 0.0539 and 0.0763 respectively (Fig. 7B). Then we estimated the relationships between miR-16 family/TFAP2A/PSG9 axis and clinical parameters in TCGA-LUAD via similarly dividing patients into miR-16 family^-^TFAP2A^+^PSG9^+^, miR-16 family^+^ TFAP2A^-^PSG9^-^ and Other group. For OS rate, we found miR-497/TFAP2A/PSG9 axis possessed significance, that is miR-497^-^ TFAP2A^+^ PSG9^+^ had the lowest OS rate, while miR-497^+^TFAP2A^-^PSG9^-^ had the highest OS rate, but miR-16/195/424 had no statistical significance (Fig. 7C). There was no obvious correlation between miR-16 family/TFAP2A/PSG9 axis and PFS rate (Fig. 7D). Besides, we found that miR-16 family/TFAP2A/PSG9 had no relationship with tumor size (Fig. 7E), but was obviously related to lymph node metastasis, that is the miR-16 family^-^ TFAP2A^+^PSG9^+^ had the highest lymph node metastasis rate, and the miR-16 family ^+^ TFAP2A^-^ PSG9^-^ had the lowest lymph node metastasis rate (except for miR-424 signal, whose lowest rate was Other group), while the miR-16 and miR-497 signals possessed statistical significance, and p-values of miR-195 and miR-424 were 0.0907 and 0.1165 (Fig. 7F). However, we found no significant relationship between miR-16 family/TFAP2A/PSG9 axis with distant metastasis (Fig. 7G), which, considering a relatively smaller numbers of patients with distant metastasis were included in the dataset, might not potently negate the pro-metastasis role of miR-16 family/TFAP2A/PSG9 axis. Therefore, the above results partially suggest the clinical effect of the miR-16 family /TFAP2A/PSG9 axis upon LUAD, especially for lymph node metastasis, and relative to miR-195/424, miR-16/497 signals might be more significant.

## Discussion

TFAP2A plays crucial roles in diverse biological processes but its function in tumors is controversial, needed to be further clarified. Herein, we demonstrated that in multiple independent datasets, TFAP2A exhibited high expression in LUAD and elevated TFAP2A levels strongly indicated poorer prognosis, heralding tumor-promoting function of TFAP2A in LUAD. However, our finding seemed contradictory with previous studies suggesting tumor-inhibition potential of TFAP2A in lung cancer. We concluded reasons could be as follows.

First, previous study mainly deduced, from lung cancer cell lines cultured in vitro, that TFAP2A could weaken some malignant phenotypes, like proliferation, apoptosis resistance, and chemotherapy insensitivity^8^. However, tumor progress, especially inside the human body, is consistently dynamic and extremely complex, involving complicated communication and reciprocal remolding between tumors and microenvironments^19,20^. Meanwhile, biological effects of specific genes differ largely according to histologic type, genetic background and environment factors, especially for pleiotropic transcriptional regulatory factors^21^. Furthermore, as a transcription factor, TFAP2A has a wide range of substrates, thus playing different roles in distinct pathways, which might be either oncogenic or tumor suppressive potential^6,7^. That is to say, one or several cell lines cultured cannot reproduce the real malignant progression situation in human body well, especially for lung cancer of highly genetic heterogeneity. And single vitro biological analysis and mediation of specific signal pathway possibly not ubiquitous and decisive might misread the real principal function of pleiotropic genes in complete carcinogenesis. However, via analyzing multiple independent clinical datasets, we have confirmed LUAD possessed broadly upregulated TFAP2A expression, which also indicted universally poorer prognosis. We have offered a relatively more objective and comprehensive assessment about clinical significance of TFAP2A upon LUAD, so we deduced that, collectively, TFAP2A could play a cancer-promoting role in LUAD malignant progression.

Further, by biological investigation in vitro and in vivo, we found TFAP2A was not prerequisite for LUAD proliferation and TFAP2A excessively exogenous overexpression even induced prohibition, however, TFAP2A could potently facilitate LUAD metastasis, possibly by triggering EMT. From our point of view, combined with previous researches, TFAP2A was not obligatory for LUAD proliferation, or even take a negative role if overly magnified, but this proliferation-suppressive effect could be counteracted by other oncogenic factors in malignant progression, only surfacing in overwhelmingly enhanced exogenous TFAP2A expression of cultured conditions. However, TFAP2A pivotally potentiated LUAD metastasis by facilitating malignant EMT, which might more conform with its biological function. General speaking, TFAP2A was a core transcriptional factors in determining embryonic development, which depends largely on EMT^5,22^. Although distinct differences exist between embryonic EMT and oncogenic EMT, core regulatory mechanisms of both have much similarities^23^. Therefore, enhanced TFAP2A in LUAD could greatly activates signals facilitating EMT, by which, epithelial tumor cells execute morphological remodeling, decrease cellular adhesion, acquire enhanced migratory and invasive ability^24^. Given that metastasis builds the leading cause for cancer-related death^25^, pro-metastatic property of TFAP2A could also account for its high correlation with poor prognosis in LUAD.

However, how does TFAP2A regulate EMT? We demonstrated further that TFAP2A could transactivate PSG9, which thereby partly undertakes the role of TFAP2A in promoting EMT. PSG9 belongs to the PSG family and functions markedly during pregnancy^26^. Studies have shown that PSG9 could possibly bind latent TGF-β to release TGF-β^17^. As is known, the activation of TGF-β pathway is the core for triggering EMT^18^. We then proved the necessity of TGF-β signaling activation in EMT promotion caused by TFAP2A-PSG9 axis. Therefore, we think that the highly expressed TFAP2A in LUAD transcriptionally activates PSG9, which ignites TGF-β pathway activation through forcing latent TGF-β to releasing TGF-β, hence, inducing EMT and malignant metastasis.

Simultaneously, we discussed the possible molecular underpinnings for abnormally upregulation of TFAP2A in LUAD. As we know, miRNAs occupy a critical position in gene expression regulation^27^. By bioinformatic prediction and subsequent validation, we proved miR-16 family (miR-16-5p/miR-195-5p/miR-424-5p/miR-497-5p) could decline TFAP2A expression by post-transcriptional silencing, but the specific sites in TFAP2A-3′UTR, which the seed region paired to, are different among members of miR-16 family. In fact, miR-16 family exhibits tumor suppressive potential in many tumors like inducing cell cycle arrest by targeting cycle dependent proteins^28,29^, and we found a novel oncogenic role of miR-16 family, that is to hamper TFAP2A from triggering EMT. We also demonstrated in several transcriptional datasets, that miR-16/195/497 exhibited low expression in LUAD, partly indicating liberation of 3′-UTR inhibition by miR-16/195/497 could account partly for enhanced TFAP2A expression in LUAD. However, we found miR-424 possessed high level in LUAD, which is indeed coherent with previous studies that miR-424 was upregulated in various cancers and exhibited tumor-promoting function, quite opposite with other miR-16 members^30–33^. This suggested us that suppression of miR-424-5p upon TFAP2A might not dominant in LUAD. Additionally, we also confirmed the cancer-regulating function of miR-16 family/TFAP2A/PSG9 axis in LUAD clinical specimens, especially for lymph node metastasis. However, the clinical significance of miR-497/16, relative to miR-195/424, seemed more significant. It also suggested that members of the miR-16 family have different weights for the regulation of TFAP2A/PSG9 axis. In fact, gene expression regulation is involved with many aspects like epigenetic modification, transcriptional or post-transcriptional regulation, translational or post-translational mediation, that is to say, regulation of miRNA-TFAP2A might be only one aspect of TFAP2A regulation. How weight of miRNA-regulation and how weight of each miRNAs all need to be clarified, which would be done in our further work.

In conclusion, we demonstrated that TFAP2A exhibited high expression in LUAD, and high TFAP2A level could be a novel prognostic risk factor for LUAD. Mechanically speaking, TFAP2A could transactivate PSG9 to enhance TGF-β-triggered EMT, thus reinforcing migration and invasion of LUAD, while suppression of miR-16 family in LUAD partly contribute to elevated TFAP2A expression through liberating inhibition of TFAP2A-3′UTR.

## Supplementary information

Legends for Supplymentary materials

Figure S1

Figure S2

Suppl. Table 1

Suppl. Table 2

Suppl. Table 3

## Data Availability

LUAD transcriptional datasets can be found in https://www.ncbi.nlm.nih.gov/geo/ and https://portal.gdc.cancer.gov/.
