# Supplementary material for: TFAP2A potentiates lung adenocarcinoma metastasis by a novel miR-16 family/TFAP2A/PSG9/TGF-β signaling pathway
Source: Cell Death Dis. 2021 Apr 6;12(4):352. doi: 10.1038/s41419-021-03606-x (PMC8024312; doi:10.1038/s41419-021-03606-x)
Supplement: Supplementary file 1 — Legends for Supplymentary materials [file 41419_2021_3606_MOESM1_ESM.docx]

**Fig. S1 TFAP2A could promote LUAD metastasis. A** Cell migratory and invasive ability (left) by transwell assay as well as statistical analysis (right) for TFAP2A overexpression cell models of PC-9. **B** Cell migratory ability via wound-healing assay (left) and statistical analysis (right) for TFAP2A overexpression cell models of PC-9. **C** Cell migratory and invasive ability (left) by transwell assay as well as statistical analysis (right) for TFAP2A knockdown cell models of H1650. **D** Cell migratory and invasive ability (left) by transwell assay as well as statistical analysis (right) for TFAP2A overexpression cell models of H1650. **E** Cell migratory ability via wound-healing assay (left) and statistical analysis (right) for TFAP2A knockdown cell models of H1650. **F** Cell migratory ability via wound-healing assay (left) and statistical analysis (right) for TFAP2A overexpression cell models of H1650. *, p <0.05; **, p<0.01; ***, p<0.001. Data are presented as mean ± SD.

**Fig. S2 TFAP2A could undergo post-transcriptional silencing by miR-16 family. A, D** Overexpression efficacy for miR-16/195/424/497-5p mimics in H1650 (**A**) and HCC827 (**D**). **B, E** Influence on TFAP2A transcript levels of miR-16/195/424/497-5p mimics transfection in H1650 (**B**) and HCC827 (**E**). **C, F** Influence on TFAP2A and PSG9 protein levels of miR-16/195/424/497-5p mimics transfection in H1650 (**C**) and HCC827 (**F**). **G** Schematic representation about WT site and five Mut sites of 3’UTR of TFAP2A, which the seeding region of miR-16 family match. *, p <0.05; **, p<0.01; ***, p<0.001. Data are presented as mean ± SD or boxplots.

**Table Suppl.**

Supplementary Table 1, LUAD datasets applied in this article

Supplementary Table 2, Primers for detecting gene transcription and sequences of nucleic acids for RNA interference

Supplementary Table 3, Primers for detecting PSG9 promoter region
