# Supplementary figures and images for: TFAP2A potentiates lung adenocarcinoma metastasis by a novel miR-16 family/TFAP2A/PSG9/TGF-β signaling pathway
Source: Cell Death Dis. 2021 Apr 6;12(4):352. doi: 10.1038/s41419-021-03606-x (PMC8024312; doi:10.1038/s41419-021-03606-x)

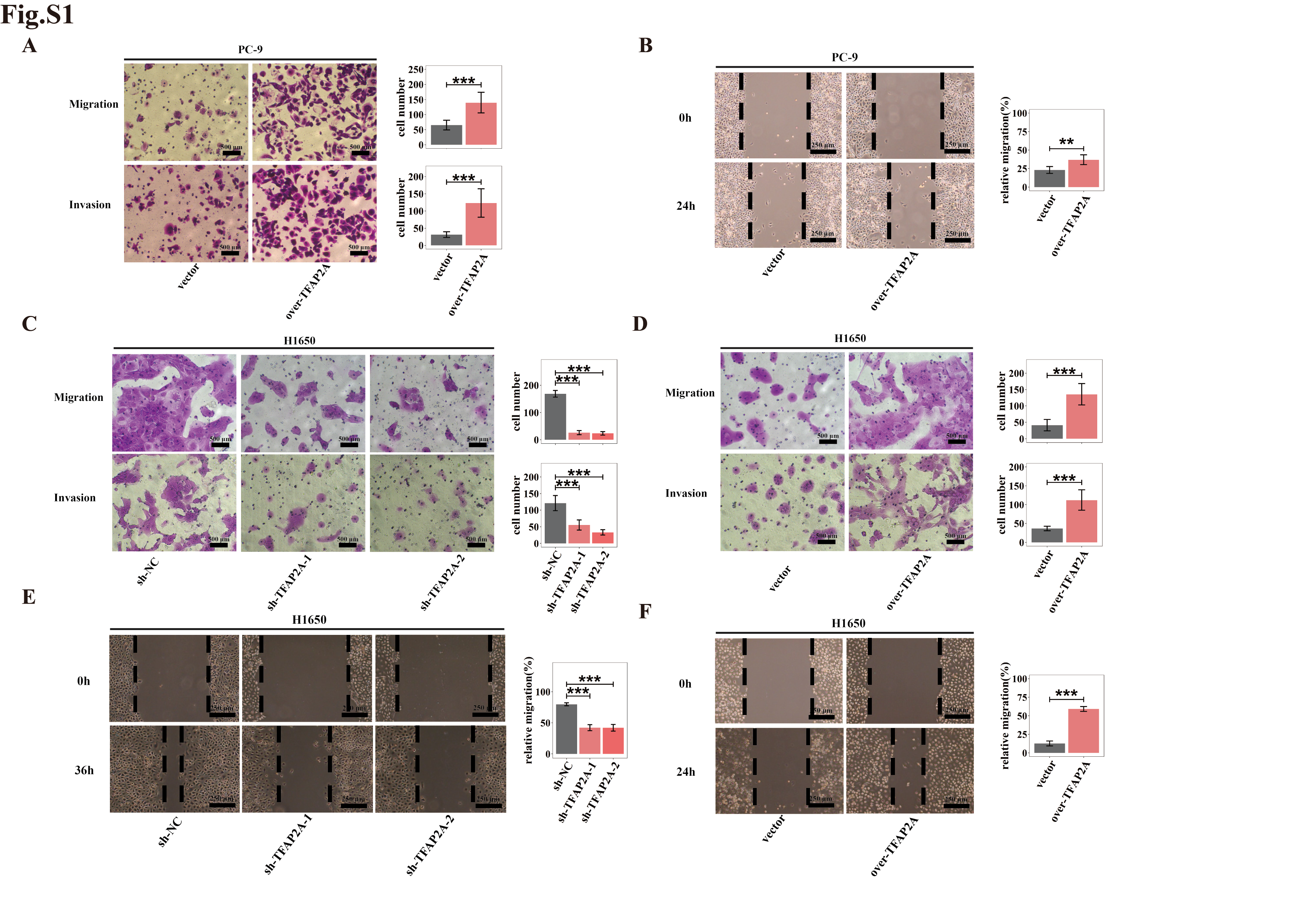

Supplement: Supplementary file 2 — Figure S1 [file 41419_2021_3606_MOESM2_ESM.png]

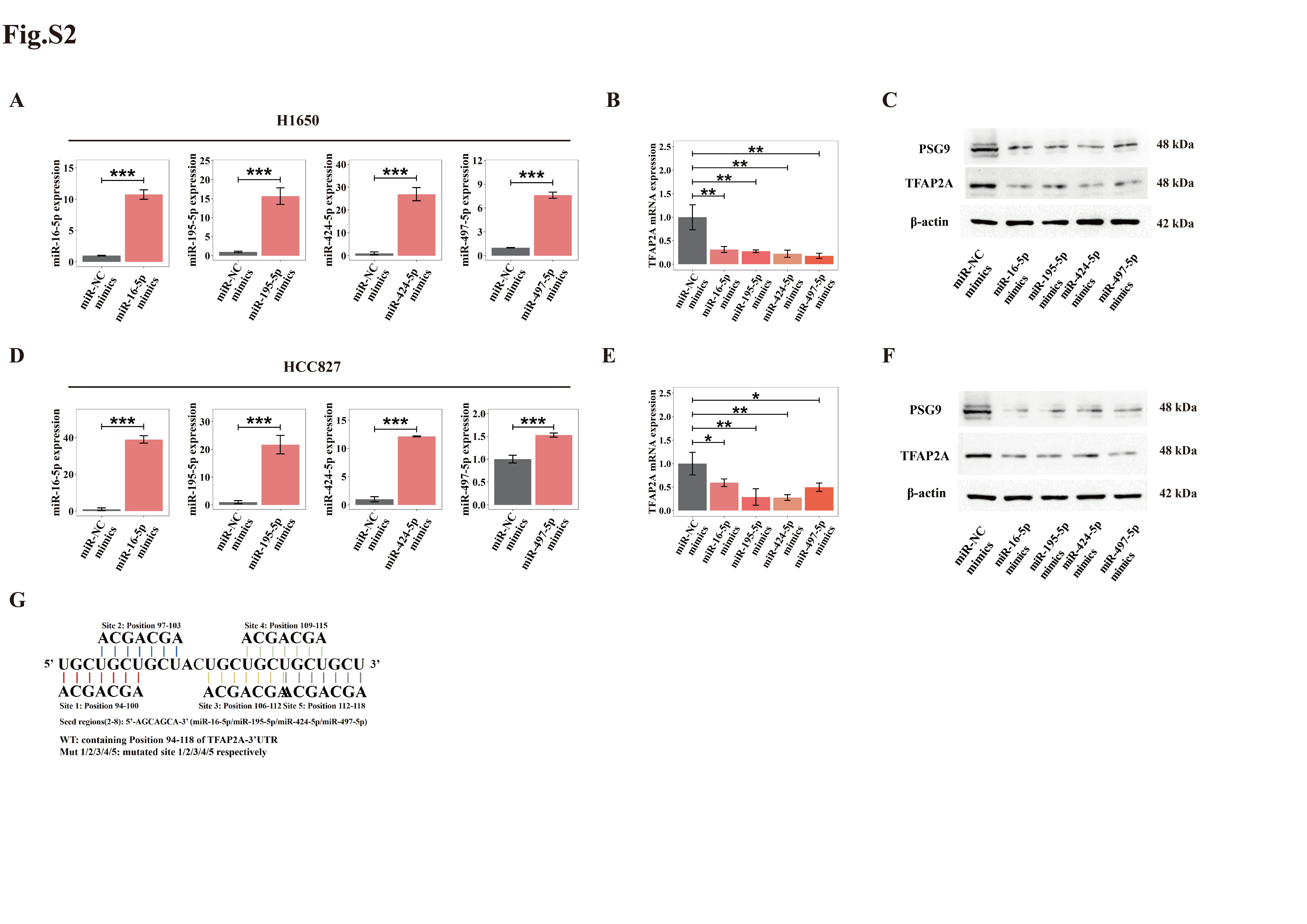

Supplement: Supplementary file 3 — Figure S2 [file 41419_2021_3606_MOESM3_ESM.png]
