# Supplementary material for: TFAP2A potentiates lung adenocarcinoma metastasis by a novel miR-16 family/TFAP2A/PSG9/TGF-β signaling pathway
Source: Cell Death Dis. 2021 Apr 6;12(4):352. doi: 10.1038/s41419-021-03606-x (PMC8024312; doi:10.1038/s41419-021-03606-x)
Supplement: Supplementary file 6 — Suppl. Table 3 [file 41419_2021_3606_MOESM6_ESM.docx]

| **Supplementary Table 3**  **Primers for detecting PSG9 promoter region** | |  |
| --- | --- | --- |
| **Region** | **Primers** |  |
| **PSG9-p1** | **forward: 5'-CCCCAGGGTATTCACAGTGTT -3' reverse: 5'-TTCACCTTCCAGCCCCGT-3'** |  |
|  |  |  |
| **PSG9-p2** | **forward: 5'-TGCAGCCTGGCCTGGGA-3' reverse: 5'-GACCCTGGTGAGGGT-3'** |  |
|  |  |  |
| **PSG9-p3** | **forward: 5'-GGCCCAAGTCCTCAGC-3' reverse: 5'-GAGATGCCTTTAGCT-3'** |  |
|  |  |  |
| **PSG9-p4** | **forward: 5'-ATCTCCCAGGGCAGCG-3' reverse: 5'-TTGGCCTGGGAAGCC-3'** |  |
|  |  |  |
| **PSG9-p5** | **forward: 5'-TCTGGGGCTTCCCAGG-3' reverse: 5'-CGGATGGTACCTGTG-3'** |  |
|  |  |  |
| **PSG9-p6** | **forward: 5'-GCTCAGAGAGCGTGT-3' reverse: 5'-GGGTGAGTTGTGTTC-3'** |  |
|  |  |  |
| **PSG9-p7** | **forward: 5'-GTCAAAACCCTCAGGA-3' reverse: 5'-GGACATGGCTCATTG-3'** |  |
|  |  |  |
| **PSG9-p8** | **forward: 5'-GTCCCAGGCTCCCCCT-3' reverse: 5'-CTGACCATCTTTGATG-3'** |  |
|  |  |  |
| **PSG9-p9** | **forward: 5'-AGCTCCAGCTCAGCC-3' reverse: 5'-CTTCCTGGAGGTGTG-3'** |  |
|  |  |  |
| **PSG9-p10** | **forward: 5'-CCCTGCACACCTCCAG-3' reverse: 5'-CCCATTGTGCTGTGG-3'** |  |
|  |  |  |
| **PSG9-p11** | **forward: 5'-CAGCCTCCTGCACTGAA-3' reverse: 5'-ACACTTTGTGCAGACA-3'** |  |
|  |  |  |
| **PSG9-p12** | **forward: 5'-ACAACTCCCAGGGACCT-3' reverse: 5'-TGCTGAGCCTCTTCCC-3'** |  |
|  |  |  |
| **Note： Whole promoter region of PSG9 gene (2000 upstream from the transcription start point) was put into JASPAR software to predict possible binding sites for TFAP2A. Then specific primers targeted at fragments (150 bp) containing each binding site were synthesized for level estimating by q-PCR, and the mean value was regarded as levels of promoter region TFAP2A bound to.** | |  |
|  |  |  |
|  |  |  |
|  |  |  |
|  |  |  |
|  |  |  |
